# Addressing the rising colorectal cancer burden in the older adult: examining modifiable risk and protective factors for comprehensive prevention strategies

**DOI:** 10.3389/fonc.2025.1487103

**Published:** 2025-02-04

**Authors:** Ke‐Jie He, Zhejun Liu, Guoyu Gong

**Affiliations:** ^1^ The Quzhou Affiliated Hospital of Wenzhou Medical University, Quzhou People’s Hospital, Quzhou, Zhejiang, China; ^2^ The Seventh Clinical College of Guangzhou University of Chinese Medicine, Shenzhen, China; ^3^ School of Medicine, Xiamen University, Xiamen, China

**Keywords:** colorectal cancer, obesity, epidemiology, socioeconomic disparities, gender differences, older adult population

## Abstract

**Background:**

Colorectal cancer is one of the most prevalent and deadly cancer types worldwide. Emerging evidence suggests that high body mass index (BMI) is a significant risk factor for colorectal cancer, particularly among the older adult population. This comprehensive analysis aims to explore the complex epidemiological patterns of colorectal cancer, with a focus on the association between high BMI and disease burden in the older adult.

**Methods:**

The study leveraged data from the Global Burden of Disease (GBD) 2021 study to examine the temporal trends, regional disparities, and the interplay of age, period, and cohort factors in shaping the global colorectal cancer landscape. Epidemiological techniques, including age-period-cohort modeling and joinpoint regression analysis, were employed to provide insights into the potential drivers of the evolving disease burden while controlling for relevant confounding factors.

**Results:**

The analysis revealed significant geographical disparities in the burden of colorectal cancer among the older adult population. Countries like Uruguay, Monaco, Croatia, Hungary, and Poland exhibited higher mortality and disability-adjusted life-year (DALY) rates, while regions like Bangladesh, Nepal, and much of Africa had relatively lower disease burden. These regional differences are likely attributable to variations in healthcare systems, access to screening and early detection programs, as well as differences in lifestyle behaviors and risk factor prevalence.

**Conclusion:**

The strong association between high BMI and colorectal cancer risk, particularly in the older adult population and among men, emphasizes the importance of comprehensive obesity management strategies as part of comprehensive cancer control efforts. Targeted interventions, such as community-based weight management programs and enhanced screening initiatives in high-risk regions, could help mitigate the disproportionate burden of colorectal cancer observed in countries like Monaco, Croatia, and Hungary. Ongoing research and multifaceted public health interventions are crucial to address the growing global burden of colorectal cancer and mitigate the disproportionate impact on vulnerable populations. Strengthening healthcare systems, improving access to quality cancer care, and promoting lifestyle modifications to reduce obesity and other modifiable risk factors should be prioritized to effectively combat this pressing public health challenge.

## Introduction

Colorectal cancer, encompassing both colon and rectum cancer, is one of the most prevalent and deadly cancer types worldwide. It is the third most commonly diagnosed cancer and the second leading cause of cancer-related deaths globally (1). The development of colorectal cancer is typically associated with the progression of precancerous polyps or growths in the lining of the colon or rectum, which can transform into malignant tumors if left undetected and untreated (2, 3). Factors such as changes in dietary patterns, sedentary lifestyles, and the increasing prevalence of obesity among old people may contribute to the observed rise in colorectal cancer incidence in this age group (4, 5).

Emerging evidence suggests that high body mass index (BMI) is a significant risk factor for colorectal cancer, particularly among the older adult population aged 70 and above (6–8). For instance, a large prospective study conducted in Norway found that higher BMI was associated with an increased risk of colorectal cancer, and this association was even stronger in the older adult population (9). The underlying mechanisms linking obesity and increased colorectal cancer risk in the older adult may involve factors such as insulin resistance, chronic inflammation, and dysregulated lipid metabolism (10, 11). These physiological changes associated with obesity can create a pro-carcinogenic microenvironment, promote genomic instability, and alter signaling pathways, thereby facilitating the development and progression of colorectal tumors. Additionally, early-life exposure to obesity and related metabolic disturbances may have long-lasting consequences, exacerbating the risk of colorectal cancer in the later stages of life9. This potential cohort effect, where the experiences and exposures of younger generations shape the disease burden in the older adult, is an important consideration in understanding the complex epidemiological patterns of this cancer.

By leveraging data from the Global Burden of Disease (GBD) study, this comprehensive analysis aims to explore the complex epidemiological patterns of colorectal cancer, with a particular focus on the association between high BMI and disease burden in the older adult population. The study examines the temporal trends, regional disparities, and the intricate interplay of age, period, and cohort factors in shaping the global colorectal cancer landscape. Additionally, the study provides insights into the potential drivers of the evolving disease burden, including population growth, aging, and epidemiological changes. These findings can inform targeted public health interventions and resource allocation strategies to address the growing burden of colorectal cancer effectively.

## Methods

### Data sources

The analysis in this study leveraged the comprehensive dataset from the Global Burden of Disease (GBD) 2021 study. The GBD is an extensive, collaborative research program led by the Institute for Health Metrics and Evaluation (IHME) at the University of Washington. Established in 1990, the GBD aims to quantify the comparative magnitude of health losses due to diseases, injuries, and risk factors by age, sex, and geography over time. The project involves a network of over 7,000 collaborators from more than 150 countries and territories, working together to compile data from a vast array of sources, including population censuses, household surveys, disease registries, and health service information systems. The most recent Global Burden of Disease study, GBD 2021, examined the global burden and strength of evidence for 88 risk factors across 204 countries and 811 subnational locations (12, 13).

The GBD employs rigorous, standardized methodologies to harmonize and analyze this expansive, multi-source data. This includes the use of sophisticated statistical modeling techniques, such as Bayesian meta-regression, to estimate disease burden and risk factor trends with a high degree of granularity and uncertainty quantification. The resulting GBD dataset provides comprehensive, comparable health metrics, including incidence, prevalence, mortality, and disability-adjusted life years (DALYs), for over 370 diseases and injuries across 204 countries and territories from 1990 to 2021.

A key focus of the analysis was the burden of colorectal cancer, as measured by disability-adjusted life years (DALYs) and mortality rates. Importantly, the study also examined the attributable burden associated with the risk factor of high body mass index (BMI). To provide a better understanding, the data was further disaggregated by gender and sociodemographic index (SDI) group, which encompasses five categories: low SDI, low-middle SDI, middle SDI, high-middle SDI, and high SDI.

### Data analysis

Spatial Analysis: We generated color-coded maps to visualize the global distribution of colorectal cancer burden, as measured by both disability-adjusted life years (DALYs) per 100,000 population and mortality rates per 100,000 population. These maps allowed us to identify regions with disproportionately high and low disease burden, highlighting the significant geographical disparities that exist.

Age-Period-Cohort Modeling: We employed advanced age-period-cohort modeling, implemented in R version 4.2.3, to explore the potential period and cohort effects shaping the epidemiological patterns of colorectal cancer burden linked to high BMI. This analytical framework enabled us to disentangle the complex interplay of age, period, and cohort factors in driving the observed trends.

The Global Burden of Disease study utilizes the DALY as a comprehensive metric to quantify the burden of disease. DALYs represent the sum of years of life lost due to premature mortality (YLLs) and years lived with disability (YLDs). This combined measure allows for the assessment of the impact of a disease or condition, taking into account both mortality and morbidity. By using DALYs, the analysis can provide a more holistic picture of the overall health burden attributed to colorectal cancer. The “net drift” metric used in the decomposition analysis represents the average annual percent change (AAPC) of a disease or health outcome that attributed to changes in population size or age structure. It captures the epidemiological changes, such as shifts in risk factor prevalence or improvements in prevention and treatment, that contribute to the observed trends in disease burden over time. Quantifying the net drift helps to disentangle the relative contributions of demographic shifts and true epidemiological transitions in shaping the colorectal cancer burden.

Joinpoint Regression Analysis: We conducted in-depth analyses of the temporal trends in colorectal cancer burden due to high BMI across different sociodemographic index (SDI) regions, including low, low-middle, middle, high-middle, and high SDI. By plotting annual trends, we were able to compare the contrasting patterns observed between high and low SDI settings, revealing important insights into the epidemiological transition. We utilized joinpoint regression analysis, performed with the Joinpoint Regression program version 5.0.2, to identify critical timepoints where the disease trend changed direction or magnitude.

### A retrospective cohort study

We conducted a retrospective cohort study at The Quzhou Affiliated Hospital of Wenzhou Medical University involving 80 colorectal cancer patients aged 70 years and above. Patients were categorized into two groups based on BMI: Normal BMI (18.5-23.9 kg/m²) and High BMI (>23.9 kg/m²). The study included patients with confirmed colorectal cancer diagnosis, complete medical records, and comprehensive clinical and pathological data, while excluding those with incomplete records, prior cancer history, or severe comorbidities. Data collection encompassed demographic information, clinical characteristics, pathological parameters, treatment details, and follow-up data. Survival analyses focused on Overall Survival (OS) and Progression-Free Survival (PFI), utilizing Kaplan-Meier survival curves, log-rank tests, and Cox proportional hazards regression. The research protocol was approved by the hospital’s institutional review board, ensuring ethical standards and patient confidentiality were maintained throughout the study.

## Results

### Spatial disparities in burden of colorectal cancer among the older adult globally

**Figure 1:**
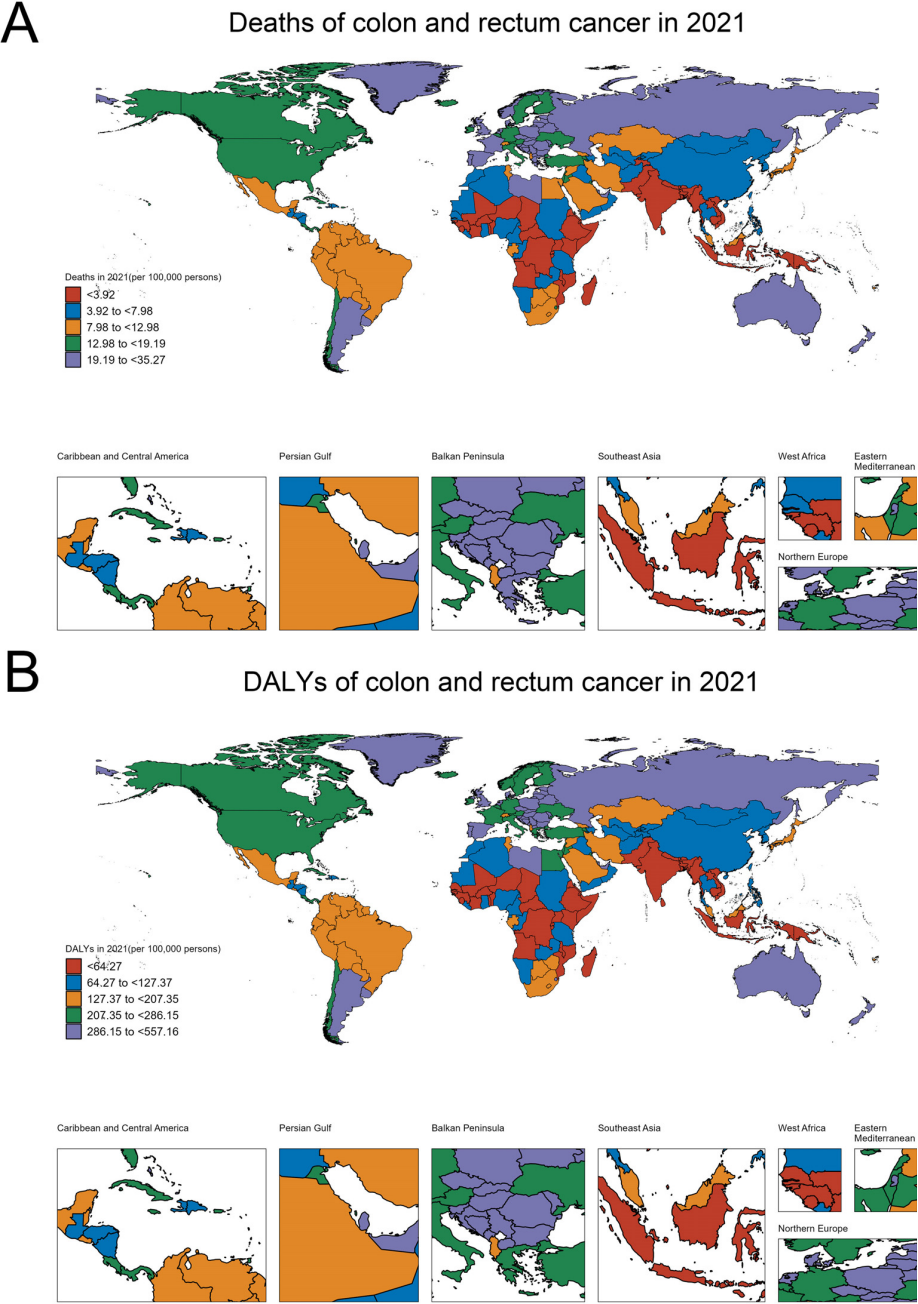
Geographical variations in the burden of colorectal cancer among the older adult population (aged ≥70 years). **(A)** Age-standardized death rates per 100,000 persons. **(B)** Age-standardized disability-adjusted life years (DALYs) per 100,000 persons.

**Figure 1A** shows the deaths of colon and rectum cancer contributed to the BMI in people over 70 years per 100,000 persons across different regions of the world. At the higher end of the spectrum, we see countries like Uruguay, Monaco, Croatia, Hungary and Poland, which have death rates ranging from 19.19 to 35.27 per 100,000 persons. In contrast, Bangladesh, Nepal, Timor-Leste, Papua New Guinea and India and most of Africa have relatively lower death rates, typically below 7.98 per 100,000 persons (**Figure 1A**).

**Figure 1B** focuses on the disability-adjusted life years (DALYs) of colon and rectum cancer contributed to the BMI in people over 70 years per 100,000 persons. The regions with the highest DALY rates are similar to those observed in the deaths map, with countries like United Arab Emirates, Croatia, Slovakia, Monaco and Hungary, exhibiting DALY rates exceeding 286.15 per 100,000 persons. On the lower end, Bangladesh, Nepal, Timor-Leste, Papua New Guinea and India have DALY rates below 64.27 per 100,000 persons (**Figure 1B**). These maps highlight the significant regional disparities in the burden of colon and rectum cancer, which can inform targeted public health interventions and resource allocation to address this cancer burden effectively.

To contextualize these geographical disparities, we examined several key factors that may contribute to the observed patterns: (1) The analysis of regional healthcare access and colorectal cancer screening rates showed that high-burden countries like Monaco, Croatia, and Hungary faced challenges in ensuring equitable access to screening services, particularly among the older adult population. In contrast, lower-burden regions such as Bangladesh and Nepal had more limited availability and utilization of early detection programs, which likely hindered the timely identification and management of colorectal cancer cases. (2) The exploration of socioeconomic factors showed that higher-income, more urbanized regions tended to have greater access to healthcare services and resources for colorectal cancer prevention and control. In contrast, lower socioeconomic status was linked to barriers in accessing screening, early diagnosis, and effective treatment, contributing to the disparities in disease burden observed across different countries and regions.

Our retrospective cohort study revealed significant differences in survival outcomes between normal and high BMI groups. The survival analyses of Overall Survival (OS) and Progression-Free Survival (PFI) demonstrated that patients in the high BMI group exhibited shorter survival times compared to those in the normal BMI group. Detailed graphical representation of these findings is provided in **Supplementary Figure 1**. These results support our earlier epidemiological observations regarding the potential impact of high BMI on colorectal cancer prognosis in the older adult population, providing clinical validation to our global burden of disease analysis.

### Temporal trends and socioeconomic disparities in the global burden of colorectal cancer

**Figure 2:**
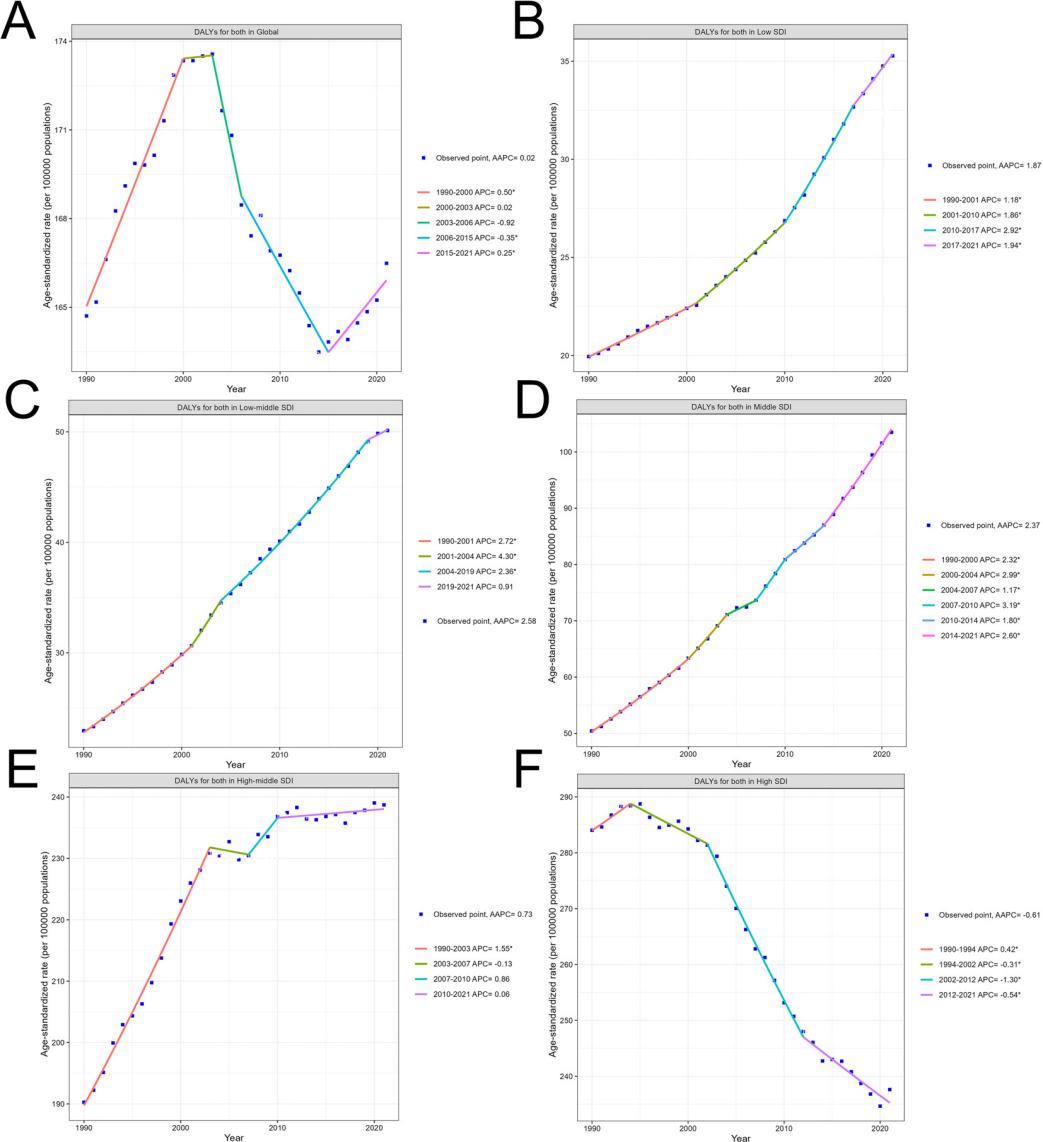
Global and country-level temporal trends in the disability-adjusted life years (DALYs) due to colorectal cancer. **(A)** Overall global trend. **(B–F)** Trends by country Socio-demographic Index (SDI) level.

**Figure 2A** shows the global trend in disability-adjusted life years (DALYs) for both sexes over time. The graph exhibits multiple joinpoints, indicating significant changes in the annual percent change (APC) over the decades. The trend starts with an increasing APC, followed by a decreasing APC, and then another increase, highlighting the complex temporal dynamics of the global disease burden. **Figures 2B–D** depict the trends in DALYs within countries categorized by their Socio-demographic Index (SDI) level. The trends show consistent increases over time, with the magnitude of change varying across the SDI groups. Countries with middle SDI generally have higher DALY rates compared to those with low SDI, low middle SDI. Turning to **Figure 2E**, this reveals a distinct pattern of disease burden change in high middle SDI countries over time. The trend starts with a rapid increase in DALYs during the early part of the time period, characterized by a high annual percent change (APC). However, the rate of increase slows down in the later years, as indicated by the decreasing APC values. This suggests that while Middle SDI countries initially experienced a substantial rise in the burden of this disease, the rate of increase has moderated in more recent decades.

In contrast, the trend observed in **Figure 2F** for high SDI countries exhibits a consistent and prolonged decrease in DALYs over the entire time period. The graph demonstrates a steady decline, with the annual percent change (APC) values remaining negative throughout since 1994.

When examining the DALY trends within countries categorized by their Socio-demographic Index (SDI) level, distinct patterns emerge. Countries with middle SDI generally have higher DALY rates compared to those with low SDI and low-middle SDI. This suggests that as countries transition to a middle level of socioeconomic development, they may face a greater burden of colorectal cancer, potentially due to changes in lifestyle factors, such as dietary patterns and physical activity, as well as variations in access to healthcare services.

Interestingly, the trend in high-middle SDI countries exhibits a unique pattern. These countries initially experienced a rapid increase in DALYs during the early part of the time period, characterized by a high APC. However, the rate of increase has slowed down in more recent decades, as indicated by the decreasing APC values. This suggests that while middle-income regions initially faced a substantial rise in the burden of colorectal cancer, the rate of increase has moderated over time. This shift may be attributed to improvements in cancer prevention, early detection, and treatment strategies implemented in these countries as they have become more economically developed. In contrast, the trend observed in high SDI countries exhibits a consistent and prolonged decrease in DALYs over the entire time period. The graph demonstrates a steady decline, with the APC values remaining negative since 1994. This indicates that high-income, more developed countries have been steadily reducing the burden of colorectal cancer, likely through their ability to invest in and implement effective cancer control measures, such as widespread implementation of screening programs and access to quality treatment.

In summary, the varying trends across different SDI groups highlight the complex interplay of socioeconomic development, healthcare infrastructure, and risk factor prevalence in shaping the epidemiological patterns of colorectal cancer. While middle-income countries initially experienced a rapid rise in the disease burden, the rate of increase has moderated in more recent decades, potentially due to improvements in cancer control efforts. In contrast, high-income nations have demonstrated a consistent and prolonged decline in the colorectal cancer burden, reflecting their greater capacity to invest in and implement comprehensive cancer prevention, early detection, and treatment strategies.

### Widening gender disparities in the epidemiological transition of colorectal cancer across socioeconomic contexts

**Figure 3:**
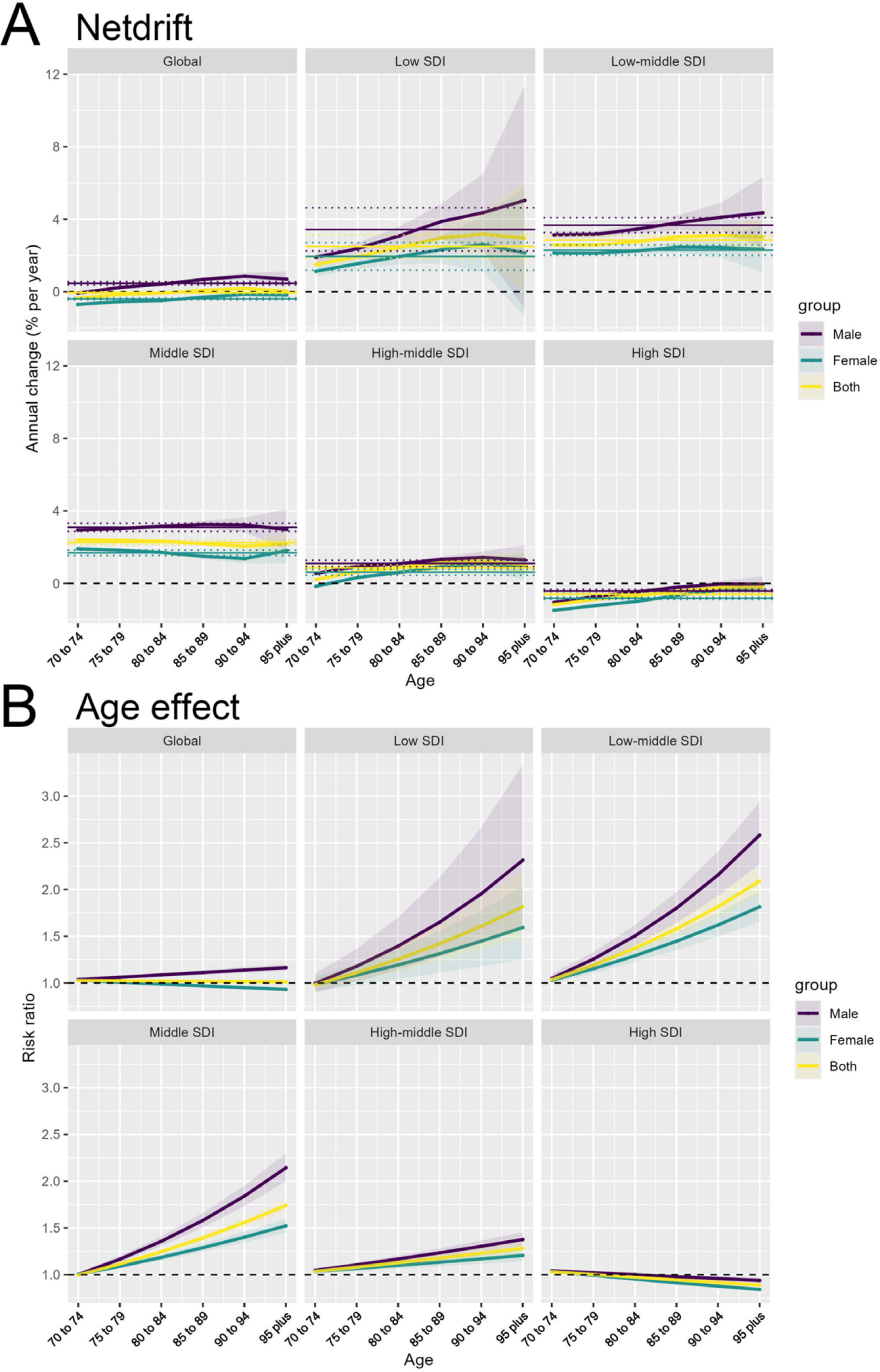
Gender disparities in the temporal and age-related patterns of colorectal cancer burden. **(A)** Net drift (Netdrift) by gender across Socio-demographic Index (SDI) groups. **(B)** Age Effect (risk ratio) by gender across SDI groups.

Across the different SDI country groups, the Netdrift is consistently higher for males compared to females. This gender disparity is observed at the global level as well as within each SDI category. When examining the SDI-specific trends, we see a clear gradient, with lower SDI countries exhibiting higher Netdrift values compared to higher SDI countries. The magnitude of the gender gap varies, with the largest differences seen in the Low and Low-middle SDI countries. In these regions, the Netdrift for males is markedly higher than for females, indicating a more pronounced temporal increase in the outcome for men. Even in the High SDI countries, where the overall Netdrift levels are lower, the male-female gap persists, suggesting that gender remains an important factor in the changing disease burden over time (**Figure 3A**). The underlying drivers of this widening gender gap may be multifactorial. Biological factors, such as hormonal differences and sex-specific metabolic pathways, may play a role in shaping the differential susceptibility and response to colorectal cancer risk factors between men and women. Additionally, gender-specific lifestyle behaviors, such as dietary patterns, physical activity levels, and healthcare-seeking practices, could contribute to the observed disparities (14). For instance, studies have shown that men tend to have higher rates of sedentary behavior and suboptimal dietary habits, which are associated with increased obesity and insulin resistance – key risk factors for colorectal cancer (15).

Similar to the Netdrift patterns, the Age Effect shows a consistent gender difference across the SDI country groups. The risk ratio for the outcome is higher for males compared to females at every age. The gender disparity is particularly pronounced in the older age groups, where the male risk ratio increases more steeply than the female risk ratio. This suggests that the age-related vulnerability to the outcome is amplified for men, especially in the later stages of life. The widening gender gap in the Age Effect is evident across the SDI spectrum, though the absolute risk levels differ, with higher SDI countries generally exhibiting lower risk ratios compared to lower SDI counterparts (**Figure 3B**). These findings highlight the importance of considering gender as a critical factor in understanding the epidemiological patterns and drivers of the disease burden. The APC analysis provides valuable insights into the complex interplay of gender, age, and socioeconomic development in shaping the temporal trends and age-specific risks.

### Birth cohort and period effects on colorectal cancer burden across socioeconomic contexts

**Figure 4:**
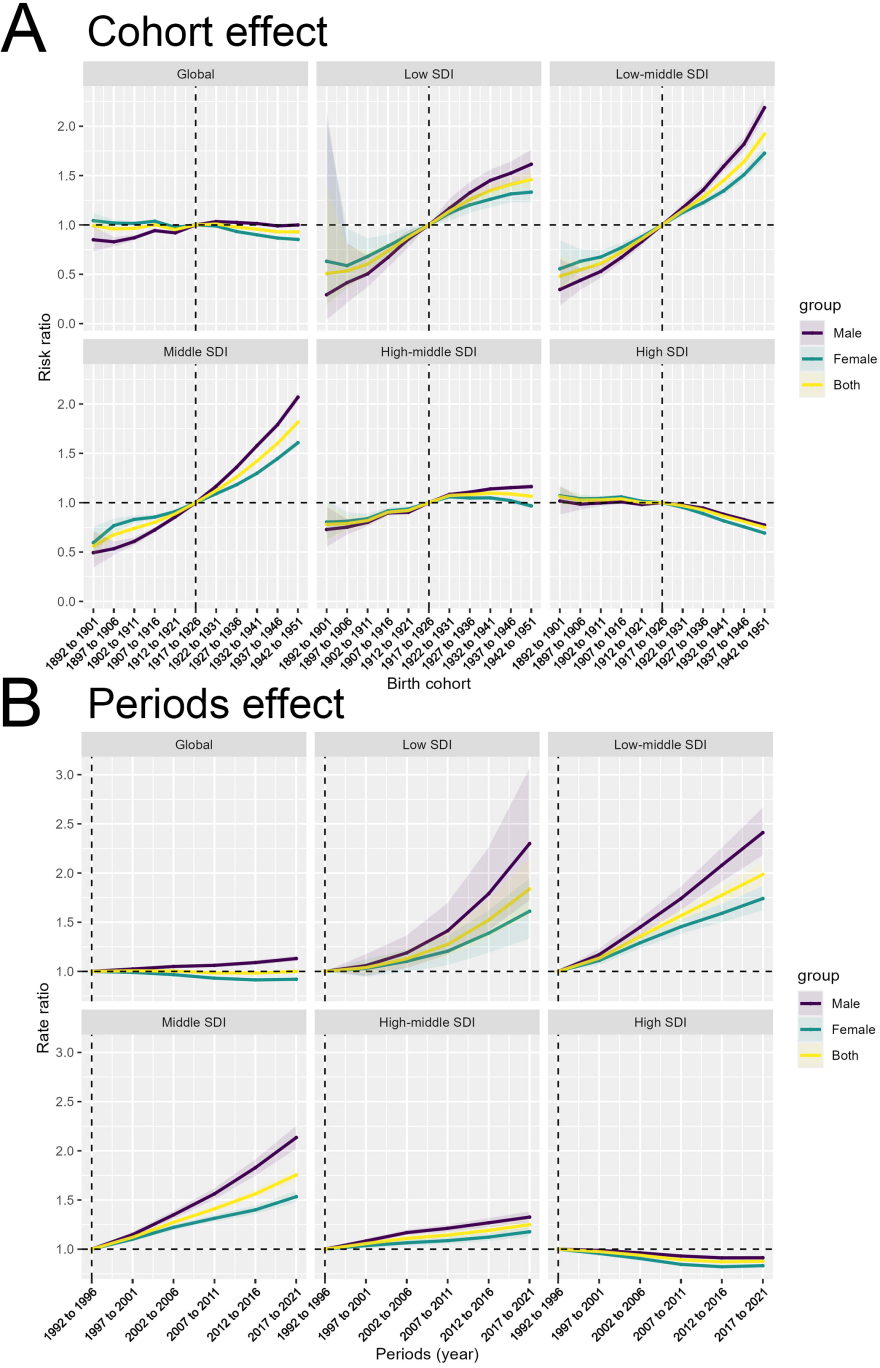
Decomposition of the temporal trends in colorectal cancer burden by birth cohort and period effects. **(A)** Birth Cohort Effect by Socio-demographic Index (SDI) level. **(B)** Period Effect by SDI level.

The Cohort Effect panels demonstrate the impact of birth cohort on the outcome across the various SDI categories. We see a clear gradient emerge at the SDI-specific trends. The Low and Low-middle SDI countries show a more pronounced increase in the Cohort Effect, with the risk ratio rising more steeply for newer birth cohorts. In contrast, for the High SDI countries, the Cohort Effect shows a consistent decreasing trend over time. This indicates that in the more developed settings, the temporal changes have actually led to a reduction in the risk ratio, likely due to improvements in prevention, early detection, and treatment of the outcome. These findings indicate that the cohort-related factors contributing to the outcome are more pronounced in less developed regions, potentially reflecting differences in factors such as environmental exposures, lifestyle behaviors, or access to preventive services across birth cohorts (**Figure 4A**).

The analysis of the epidemiological trends across different SDI country groups also revealed distinct cohort effects that may have contributed to the observed disparities in colorectal cancer burden. In high SDI countries, the consistent and prolonged decrease in disability-adjusted life years (DALYs) over the entire time period suggests that public health interventions and policies implemented in these regions have been effective in reducing the disease burden across successive birth cohorts. For instance, the widespread adoption of organized colorectal cancer screening programs, such as fecal occult blood testing and colonoscopy, has been a key strategy in high SDI countries. These screening initiatives have enabled the early detection and removal of precancerous polyps, thereby preventing the development of invasive colorectal cancer. Additionally, improvements in access to quality healthcare, including timely diagnosis and effective treatment, may have contributed to the sustained decline in the disease burden among successive cohorts in high SDI settings. In contrast, low and middle SDI countries have faced significant challenges in implementing comprehensive cancer control measures, leading to persistent disparities in colorectal cancer outcomes across cohorts. Limited access to screening services, fragmented healthcare systems, and socioeconomic barriers may have hindered the implementation of effective public health interventions in these regions. As a result, the disease burden has remained higher and increased more rapidly over time in these settings compared to the high SDI counterparts.

The Periods Effect illustrate the temporal changes in the outcome independent of age and birth cohort influences. The SDI-specific trends reveal diverging patterns. Low and Low-middle SDI countries demonstrate a more pronounced increase in the Periods Effect, suggesting that temporal factors, such as changes in social, environmental, or healthcare conditions, have had a greater impact on the outcome in these regions. Conversely, for the High SDI countries, the Cohort Effect shows a consistent decreasing trend over time (**Figure 4B**). These findings highlight the complex interplay of cohort, period, and age-related factors in shaping the epidemiological patterns of the outcome across different socioeconomic contexts.

### Decomposing the drivers of global and socioeconomic disparities in colorectal cancer burden

**Figure 5:**
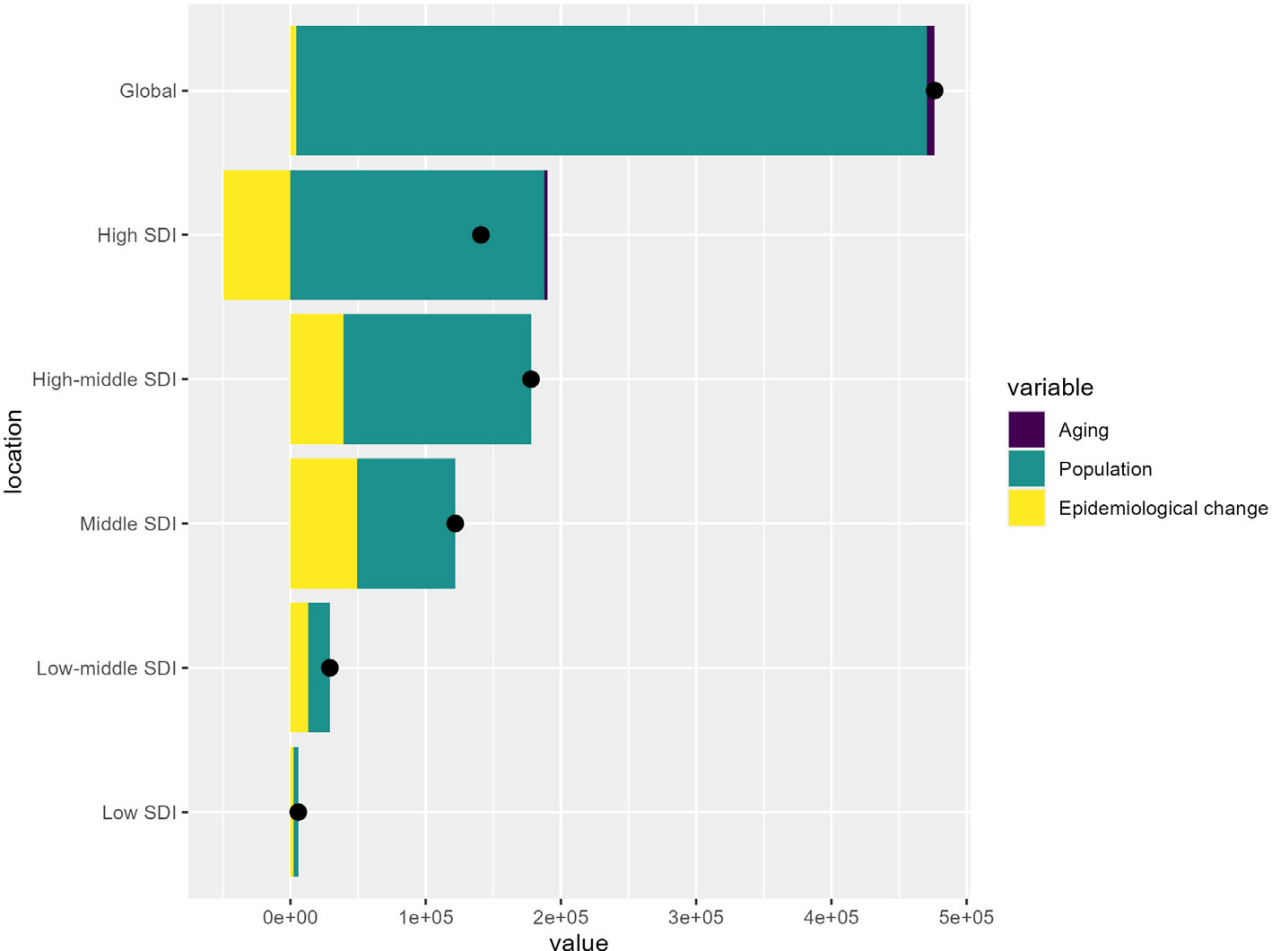
Decomposition of the changes in colorectal cancer burden from 1990 to 2021 by Socio-demographic Index (SDI) level.

**Figure 5** presents the results of a decomposition analysis, which aims to quantify the drivers of changes in a disease burden indicator over time. The vertical axis represents the Socio-demographic Index (SDI) groups, ranging from Low SDI to High SDI at the global level, while the horizontal axis shows the cumulative change in the disease burden indicator between 1990 and 2021.

At the global level, the analysis reveals that the overall increase in the disease burden was driven primarily by population growth, with a relatively smaller effect from aging and epidemiological changes. However, the contributions of these drivers vary across the different SDI groups. Low SDI countries tend to show a smaller overall increase, while high SDI countries exhibit larger overall increases, with population growth and epidemiological changes contributing more significantly.

### Forecasting the escalating burden of colorectal cancer: insights from projected incidence and disability-adjusted life years by gender and age

**Figure 6:**
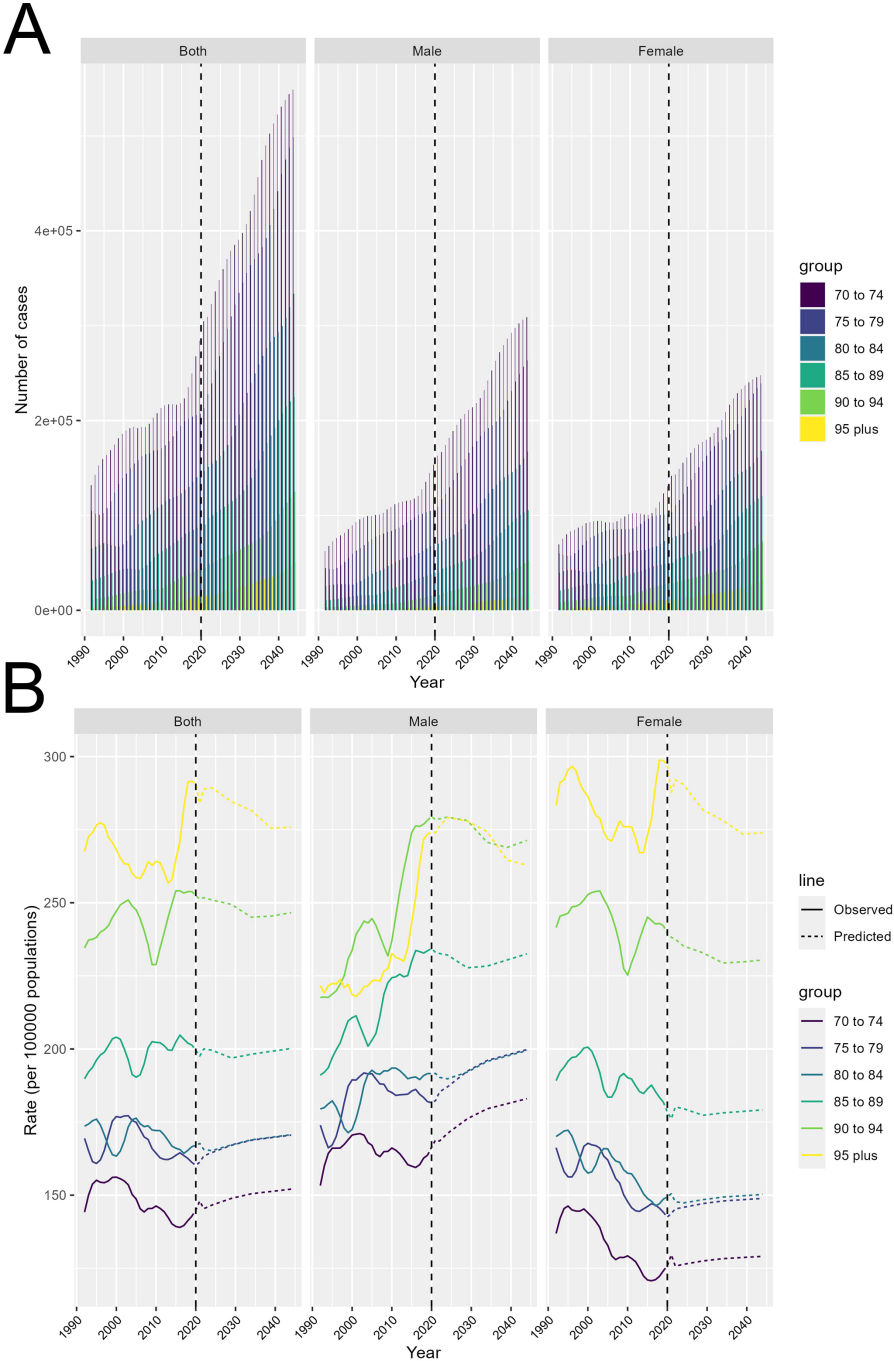
Projected trends in colorectal cancer burden from 2022 to 2044. **(A)** Total number of incident cases by age and gender. **(B)** Disability-adjusted life years (DALYs) rate by age and gender.

**Figure 6** presents projections of the number of colorectal cancer cases and DALYs rate from 2022 to 2044, stratified by gender and age group. **Figure 6A** show the projected total number of colorectal cancer cases over the years. The key observation is that the total number of cases is expected to increase significantly over this time period. The increases are most pronounced in the older age groups, with those aged 70 to 74 exhibiting the steepest projected rises. This suggests that the burden of colorectal cancer will continue to grow, particularly among the older adult population. Additionally, males are projected to have a consistently higher number of cases compared to females across all age groups, indicating a persistent gender disparity in colorectal cancer incidence.

Transitioning to **Figure 6B**, this section displays the projected DALYs rate trends over time. The solid lines represent the observed values, while the dashed lines indicate the predicted values. The gender differences are particularly noteworthy. Males are generally projected to exhibit higher DALYs rate compared to females, and this disparity becomes more pronounced in the older age groups. This suggests that older men may be at a higher risk of developing colorectal cancer compared to their female counterparts.

## Discussion

Colorectal cancer, which encompasses both colon and rectum cancer, is a major public health concern worldwide. Current evidence suggests that high body mass index (BMI) is a significant risk factor for colorectal cancer, particularly among the older adult population aged 70 and above (9, 16). The mechanism underlying the association between high BMI and increased colorectal cancer risk in the older adult may be related to factors such as insulin resistance, chronic inflammation, and dysregulated lipid metabolism (17, 18). These physiological changes associated with obesity can promote the development and progression of colorectal tumors by inducing genomic instability, altering signaling pathways, and creating a pro-carcinogenic microenvironment (19, 20).

The global epidemiology of colorectal cancer is characterized by stark inequities across different regions. These marked disparities in the geographical distribution of colorectal cancer underscore the need for targeted, context-specific public health strategies and resource allocation. It is important to note that the relationship between obesity and colorectal cancer risk may be more complex and multifactorial than a direct causal link. While the data suggests a strong association between high BMI and colorectal cancer burden, particularly in the older adult population, it is possible that obesity may correlate with other important risk factors in these high-burden regions, rather than directly driving the cancer incidence. For instance, lifestyle behaviors such as physical inactivity, unhealthy dietary patterns, and sedentary lifestyles may be more prevalent in areas with high obesity rates. These confounding factors could independently contribute to the development and progression of colorectal cancer, beyond the direct effects of excess body weight. Additionally, reverse causality should be considered, where the observed geographical disparities may be influenced by factors that predispose certain populations to both obesity and colorectal cancer, rather than obesity itself being the primary driver of the cancer burden. Socioeconomic status, access to healthcare, and cultural factors, for example, could shape both the prevalence of obesity and the ability to detect and manage colorectal cancer effectively. Genetic predispositions and family history are also important considerations, as these factors can influence an individual’s susceptibility to colorectal cancer, irrespective of their BMI status. The complex interplay between genetic, environmental, and behavioral determinants must be taken into account when interpreting the observed associations. In addition, A recent systematic review and meta-analysis by Carlotta De Filippo et al. (2024) examined the differences in gut microbiome composition between colorectal cancer patients and healthy controls (21). The study found that the relative abundance of certain bacterial genera, such as Bacteroides, Fusobacterium, and Prevotella, was significantly altered in colorectal cancer patients, suggesting a potential role of the gut microbiome in colorectal carcinogenesis. Furthermore, several studies have specifically investigated the changes in the gut microbiome that occur with aging and their implications for colorectal cancer risk. For instance, a study by Claesson et al. (2012) observed that the gut microbiota of older adult individuals (> 65 years) was less diverse and had a higher proportion of potentially pathogenic bacteria compared to younger adults (22). These age-related shifts in the gut microbial community could create a pro-inflammatory environment and disrupt the normal intestinal homeostasis, thereby increasing susceptibility to colorectal cancer.

Delving into the temporal trends, the data reveals the complex dynamics underlying the evolving global colorectal cancer burden. The overall trend displays multiple critical timepoints, indicating significant changes in the annual percent change (APC) of DALYs across the decades. This complexity highlights the value of employing advanced epidemiological techniques to identify these pivotal junctures and elucidate the drivers shaping these temporal patterns. The interplay between socioeconomic development and the colorectal cancer burden offers valuable insights. High middle SDI countries initially experienced a substantial rise in DALYs, but the rate of increase has moderated in more recent decades, suggesting that economic growth and development may be accompanied by improvements in cancer prevention, early detection, and treatment strategies. For example, China has seen a steady decline in colorectal cancer mortality rates since the early 2000s, likely due to the expansion of healthcare access and the implementation of screening programs (23, 24). In contrast, high SDI countries have demonstrated a consistent and prolonged decrease in the disease burden, likely attributable to their greater investment in effective cancer control measures (25). To address the disproportionate burden observed in high-middle SDI countries during the initial period of rapid increase, targeted interventions should be implemented. The WCRF Expert Panel’s review provides strong evidence that promoting lifestyle modifications to address key modifiable risk factors could significantly impact the colorectal cancer burden. Specifically, being physically active, consuming whole grains, dietary fiber, and dairy products can decrease colorectal cancer risk, while factors like obesity, red and processed meats, and alcohol consumption increase risk (26). By directly targeting these evidence-based risk factors through community-based weight management programs and education initiatives on healthy diets rich in whole grains and fiber, the proposed interventions can help mitigate the rising colorectal cancer burden observed in high-middle SDI countries. In low and low-middle SDI regions, the data suggests that challenges in ensuring equitable access to screening services have hindered the timely identification and management of colorectal cancer cases. In these settings, strengthening healthcare systems and improving the availability and utilization of early detection programs should be prioritized as part of comprehensive cancer control efforts. The WCRF Expert Panel’s findings emphasize that increased screening and early detection are effective strategies for reducing colorectal cancer risk, which can be particularly impactful in these lower-resource settings (27, 28). One successful example of a community-based obesity management program is the “Healthy Eating and Lifestyle Program” (HELP) implemented in Mexico. HELP targeted low-income communities and integrated nutrition education, physical activity promotion, and behavioral counseling to support sustainable lifestyle changes. Over a 3-year period, the program demonstrated significant reductions in obesity prevalence and waist circumference among participants, which could translate to a lower colorectal cancer risk in these high-risk populations (29). By directly targeting these evidence-based risk factors through community-based weight management programs, such as the HELP initiative in Mexico, the proposed interventions can help mitigate the rising colorectal cancer burden observed in high-middle SDI countries. The “Screen for Life” program in India provides a successful model for enhancing colorectal cancer screening in resource-limited regions. This community-based initiative leveraged mobile health units and trained local healthcare workers to conduct affordable, non-invasive fecal occult blood tests, enabling early detection and timely referrals for further diagnosis and treatment. Pilot studies have demonstrated increased screening rates and improved access to care, particularly among underserved populations (30).

Interestingly, the link between high BMI and colorectal cancer risk appears to be more pronounced in older adult men compared to older adult women (31). These findings align with existing literature, suggesting that gender-specific risk factors, such as hormonal differences, lifestyle behaviors, and inequitable access to healthcare, may contribute to the observed disparities (32). Hormonal factors, such as the imbalance between estrogen and androgen levels, may play a crucial role in modulating the impact of obesity on colorectal carcinogenesis (31). Emerging evidence suggests that hormonal differences between men and women play a significant role in modulating the risk and development of colorectal cancer. Studies have shown that estrogen, the primary female sex hormone, may have a protective effect against colorectal cancer by influencing cell proliferation, apoptosis, and inflammatory pathways (33). Conversely, higher levels of androgens (male sex hormones) have been associated with an increased risk of colorectal cancer, particularly among men. The androgenic influence on factors such as insulin resistance, chronic inflammation, and altered lipid metabolism may create a more permissive environment for colorectal tumor initiation and progression (34). Furthermore, the gender-specific hormonal changes that occur throughout the lifespan, such as the menopausal transition in women, may contribute to the widening gap in colorectal cancer risk between men and women, especially in the older age groups. The post-menopausal decline in estrogen levels in women may diminish the protective effects, potentially leading to a narrowing of the gender disparity in the later stages of life.

The decomposition analysis provides valuable insights into the underlying drivers of the global and socioeconomic disparities in colorectal cancer burden. At the global level, the analysis reveals that the overall increase in the disease burden was primarily driven by population growth, with a relatively smaller effect from aging and epidemiological changes. However, the contributions of these drivers vary significantly across the different SDI groups. Low SDI countries tend to show a smaller overall increase in colorectal cancer burden, while high SDI countries exhibit larger overall increases. This divergence may be attributed to several factors: (1) Demographic transitions: Low SDI countries are still undergoing demographic transitions, with younger age structures and slower population aging compared to high SDI nations. As a result, the impact of population growth on the colorectal cancer burden is more pronounced in high SDI settings, where the population is aging more rapidly. (2) Risk factor prevalence: High SDI countries have experienced more substantial changes in lifestyle factors, such as diet, physical activity, and obesity, which are key risk factors for colorectal cancer. The epidemiological transition in these regions has likely contributed to a larger increase in the disease burden compared to low SDI settings, where risk factor profiles may be relatively more stable. (3) Healthcare access and cancer control measures: High SDI countries generally have more advanced healthcare systems and have been able to implement comprehensive cancer control strategies, including widespread screening programs and improved access to early diagnosis and treatment. However, these interventions may not have been as effectively implemented in low SDI regions, leading to a smaller overall increase in the disease burden. (4) Socioeconomic and environmental influences: Factors such as income inequality, education levels, and environmental exposures can vary significantly between high and low SDI countries. These socioeconomic and environmental determinants may shape the epidemiological patterns and contribute to the disparities in colorectal cancer burden across the SDI spectrum.

The rising prevalence of obesity, a well-established risk factor for colorectal cancer, has been a key driver of the growing disease burden, particularly among the older adult population. As the global population continues to age, the absolute number of colorectal cancer cases and related deaths is expected to increase substantially. However, it is important to distinguish between the absolute increases in disease burden and trends in age-standardized rates, which account for changes in the underlying population structure. While the total number of colorectal cancer cases may rise due to population aging, the age-standardized incidence and mortality rates could tell a different events. Examining the age-standardized epidemiological trends is crucial to understanding the true progression of the disease burden, independent of demographic shifts. This distinction is important to prevent overestimating the problem and to ensure that public health interventions are appropriately targeted and effective. Comprehensive strategies to address the growing colorectal cancer epidemic in the older adult should focus on both primary prevention, targeting modifiable risk factors like obesity, as well as early detection through enhanced screening programs. Tailoring these interventions to the specific needs and challenges of the aging population will be crucial to mitigate the disproportionate burden of this disease in the later stages of life.

The complex interplay of age, period, and cohort factors in shaping the epidemiological patterns of colorectal cancer is further elucidated by the Cohort Effect and Periods Effect analyses. The divergent trends across SDI groups indicate that the relative importance of these factors may vary depending on the socioeconomic context (35, 36). For instance, less developed regions may experience more pronounced cohort-related changes, potentially reflecting differences in environmental exposures, lifestyle behaviors, or access to preventive services across birth cohorts (32, 37). The colorectal cancer burden has increased more rapidly among older cohorts, potentially due to the adoption of Westernized diets and sedentary lifestyles (9, 16, 38). Furthermore, the impact of high BMI on colorectal cancer risk becomes increasingly significant with advancing age. As individuals grow older, the cumulative effects of genetic, environmental, and lifestyle factors, coupled with the natural decline in physiological functions, may amplify the influence of obesity on the development of colorectal tumors (39, 40). Older adults may also have a reduced capacity to metabolize and eliminate carcinogenic compounds, rendering them more susceptible to the deleterious effects of obesity (41, 42).

The analysis reveals that the total number of colorectal cancer cases is expected to increase significantly in the coming years, particularly among the older adult population. However, it is important to note that this projected increase may be partially driven by improved screening and detection, rather than solely reflecting a rise in the underlying incidence of the disease. Enhanced colorectal cancer screening programs, such as the implementation of fecal occult blood testing and colonoscopy, have enabled the earlier identification of precancerous polyps and early-stage cancers in many high-income countries. This has likely contributed to the observed increases in reported cases, as more individuals are being diagnosed through these proactive screening efforts. At the same time, the analysis also indicates that the disease burden, as measured by disability-adjusted life years (DALYs), is projected to continue rising, particularly among older males. This suggests that while improved screening has led to increased case detection, the actual incidence and severity of colorectal cancer may also be escalating, especially in vulnerable populations. It will be important for future research to further disentangle the relative contributions of enhanced screening and true incidence changes in driving the observed trends. Careful monitoring of both case detection and disease burden metrics will be crucial to guide the development and implementation of targeted interventions to effectively combat the growing colorectal cancer epidemic, particularly among the older adult.

These findings underscore the importance of targeted interventions for weight management, particularly in the older adult population. The strong association between high BMI and colorectal cancer risk, particularly in the older adult population and among men, emphasizes the importance of comprehensive obesity management strategies as part of comprehensive cancer control efforts. Targeted interventions, such as community-based weight management programs and enhanced screening initiatives in high-risk regions, could help mitigate the disproportionate burden of colorectal cancer. For example, the HELP PD study, a community-based lifestyle intervention program, has demonstrated significant success in achieving and maintaining weight loss among older adults with obesity (43). The program combines dietary modifications, increased physical activity, and behavioral therapy, delivered through group sessions and individual counseling. By addressing the unique needs and barriers faced by the older adult population, such targeted programs can effectively promote healthy weight management and ultimately reduce the risk of obesity-related complications, including colorectal cancer. Furthermore, the Stepped Care approach to obesity management, implemented in the UK, provides a tiered model of interventions tailored to the individual’s needs and preferences (44). This approach starts with brief advice and self-management support, progressing to more intensive lifestyle programs, pharmacotherapy, and ultimately, bariatric surgery for those with severe obesity.

Addressing the growing burden of colorectal cancer will require a multifaceted approach that tackles the obesity epidemic, particularly among vulnerable populations. Drawing on the insights from recent studies, prevention programs targeting obesity should be a key priority. For instance, the European World Health Organization member states have implemented various initiatives to fight obesity in children, including school-based interventions, family-focused programs, and policies to promote healthy lifestyles (45). These comprehensive, multilevel approaches addressing both individual behaviors and environmental determinants have shown promise in reducing childhood obesity rates and may have long-lasting impacts on reducing colorectal cancer risk in later life. Furthermore, the workplace has been identified as a crucial setting for obesity prevention and management. Studies have highlighted the higher prevalence of overweight and obesity among specific occupational groups, such as seafarers working on merchant ships (46). Workplace-based health promotion programs that encourage physical activity, healthy eating, and support for weight management could be an effective strategy to target populations at higher risk of obesity-related cancers, including colorectal cancer (47). Integrating such a comprehensive, personalized strategy within the healthcare system can improve access to effective weight management services, particularly for the older adult population at high risk of colorectal cancer.

It is important to acknowledge the potential limitations of drawing conclusions about individual-level risk from the population-level data used in this analysis. The ecological fallacy, which refers to the error of inferring individual-level associations from group-level data, is a key consideration. While the study has revealed a strong association between high BMI and the burden of colorectal cancer at the population level, particularly among the older adult, individual risks may vary due to genetic factors, lifestyle behaviors, and other confounding variables. The strong association between high BMI and increased colorectal cancer risk, particularly in the older adult population and among men, underscores the importance of comprehensive obesity management strategies as part of comprehensive cancer control efforts. However, it is important to note that the relationship between BMI and colorectal cancer is complex and influenced by various confounding factors. Dietary patterns, physical activity levels, and genetic predispositions can all independently contribute to an individual’s colorectal cancer risk, irrespective of their BMI status. For instance, studies have shown that a diet high in processed meats and alcohol can increase the risk of colorectal cancer, even among individuals with a healthy BMI (48). Conversely, regular physical activity and a diet rich in fiber, whole grains, and dairy products can lower the risk, even in individuals with overweight or obesity (49). Nonetheless, the observed epidemiological patterns provide valuable insights to guide public health interventions targeting the modifiable risk factors contributing to the global burden of this disease. It is important to note that the GBD dataset, while comprehensive, may be subject to potential biases and limitations inherent in the underlying data sources. Specifically, regional variations in cancer registry coding practices and data collection methods could affect the accuracy and comparability of colorectal cancer burden estimates across different countries and territories. While the GBD study employs standardized methodologies to harmonize and adjust the data, residual differences in cancer registration and reporting may still influence the observed geographical disparities. Therefore, the interpretation of the results should be made with an awareness of these potential limitations in the data sources.

## Conclusion

The strong association between high BMI and colorectal cancer risk, particularly in the older adult population and among men, emphasizes the importance of comprehensive obesity management strategies as part of comprehensive cancer control efforts. Targeted interventions, such as community-based weight management programs and enhanced screening initiatives in high-risk regions, could help mitigate the disproportionate burden of colorectal cancer. However, the feasibility and cost-effectiveness of these interventions must be carefully considered, especially in resource-limited, low-SDI settings. Implementing intensive, specialized weight management programs may face challenges in terms of accessibility, affordability, and sustainability for vulnerable populations. In these contexts, a more pragmatic approach may involve integrating obesity prevention and management strategies into primary healthcare systems, leveraging existing infrastructure and community-based resources. For example, the WHO’s “Best Buys” for noncommunicable disease prevention recommend the implementation of population-wide awareness campaigns, taxation on unhealthy foods and beverages, and the provision of basic obesity counseling within primary care. These low-cost, scalable interventions can be tailored to local needs and resources, making them more feasible for adoption in low-SDI regions. Coupling these population-level strategies with targeted screening initiatives and equitable access to colorectal cancer diagnosis and treatment will be crucial to addressing the growing burden of this disease across socioeconomic divides.

## Data Availability

The original contributions presented in the study are included in the article/**Supplementary Material**. Further inquiries can be directed to the corresponding author.
