# Supplementary figures and images for: Addressing the rising colorectal cancer burden in the older adult: examining modifiable risk and protective factors for comprehensive prevention strategies
Source: Front Oncol. 2025 Feb 4;15:1487103. doi: 10.3389/fonc.2025.1487103 (PMC11841409; doi:10.3389/fonc.2025.1487103)

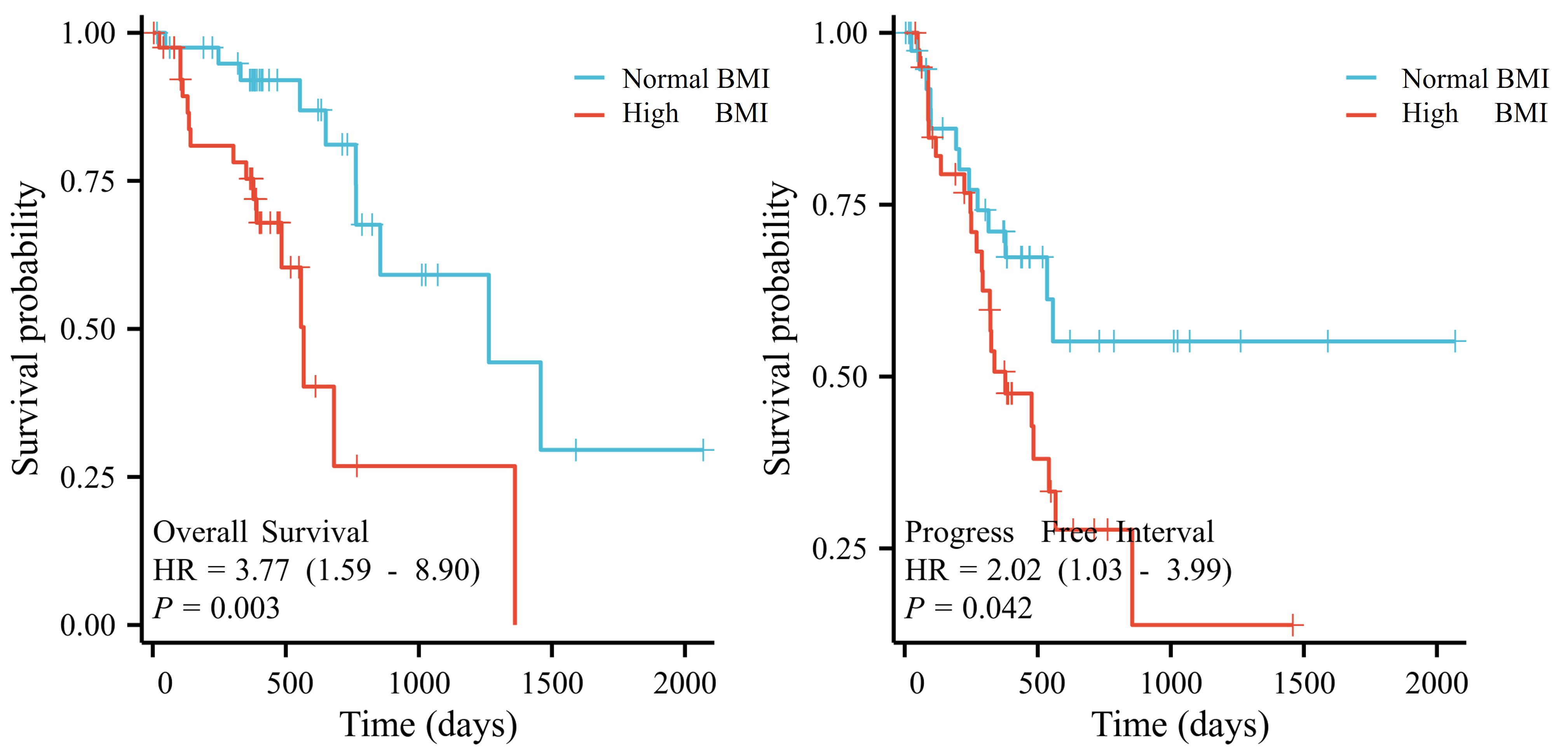

Supplement: Supplementary file 1 [file Image1.jpeg]
